# Mechanisms of p53 Functional De-Regulation: Role of the IκB-α/p53 Complex

**DOI:** 10.3390/ijms17121997

**Published:** 2016-11-29

**Authors:** Giovanna Carrà, Sabrina Crivellaro, Riccardo Taulli, Angelo Guerrasio, Giuseppe Saglio, Alessandro Morotti

**Affiliations:** 1 Department of Clinical and Biological Sciences, University of Turin, Regione Gonzole 10, 10043 Turin, Italy; gio.sax2010@hotmail.it (G.C.); sabrina.crive@hotmail.it (S.C.); angelo.guerrasio@unito.it (A.G.); giuseppe.saglio@unito.it (G.S.); 2 Department of Oncology, University of Turin, Regione Gonzole 10, 10043 Turin, Italy; riccardo.taulli@unito.it

**Keywords:** p53, IκB-α, tumor suppressors, functional inactivation

## Abstract

*TP53* is one of the most frequently-mutated and deleted tumor suppressors in cancer, with a dramatic correlation with dismal prognoses. In addition to genetic inactivation, the p53 protein can be functionally inactivated in cancer, through post-transductional modifications, changes in cellular compartmentalization, and interactions with other proteins. Here, we review the mechanisms of p53 functional inactivation, with a particular emphasis on the interaction between p53 and IκB-α, the *NFKBIA* gene product.

## 1. Introduction

**Figure 1:**
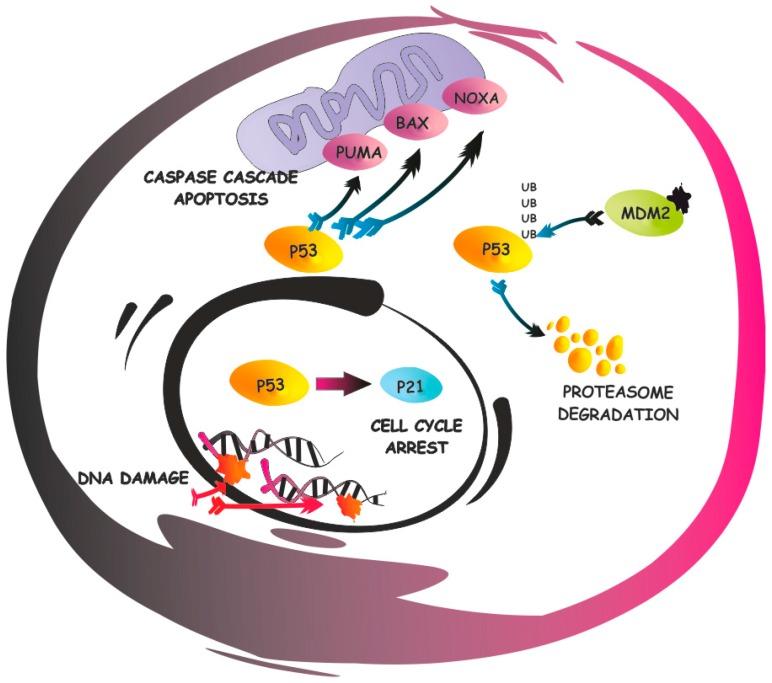
p53 pathway. A simplified representation of the apoptotic signaling pathway and p53 negative regulation by E3 ubiquitin-protein ligase Mdm2. Under stress conditions, enhanced p53 activity promotes transcription of downstream targets, such as p21, which trigger cell cycle arrest or induce cellular apoptosis. Ub: ubiquitin; PUMA: p53 upregulated modulator of apoptosis; BAX: Bcl-2-associated X protein; NOXA: phorbol-12-myristate-13-acetate-induced protein 1 (also known as *PMAIP1*). Activation or “transition-states” such as phosphorilation or ubiquitination, are shown in arrowhead; Inhibition is represented as an arrow with transversal bar.

*TP53* is the most renowned tumor suppressor among clinicians and researchers, and is one of the most extensively studied [1,2,3,4]. Its fame arises from the impressive frequency of *TP53* genetic inactivation in about 50% of cancers [5,6], the dismal association with prognoses [7,8], and the ability to regulate cellular fate [9] (Figure 1). While the role of p53 in cancer has mostly been associated with the genetic inactivation of *TP53* through mutations and deletions [1,10], various novel p53 functions and targets have been discovered, with impressive consequences in tumorigenesis [1,11,12] and cancer therapy [13,14,15,16]. Indeed, the role of p53 in cancer should include the following, novel, non-canonical functions: (i) various *TP53* mutations behave as gain of functions [1,17,18,19,20]; (ii) the p53 protein is no longer just a transcriptional factor [21,22,23,24], but acts in different cellular compartments outside the nucleus, where it mediates several processes through a complex network of partners [25,26,27,28]; (iii) wild-type p53 should also result in being functionally inactive through changes in compartmentalization or protein modifications [29,30,31]; and (iv) non-cell-autonomous tumor suppression properties of p53 have also been discovered [32]. All together, these intriguing p53 functions suggest that the role of p53 in cancer is much more complex and may be exploited from a therapeutic standpoint [15,33,34]. It is, indeed, clear that the restoration of p53 in various cancer models is responsible for cancer eradication [35,36], however, unfortunately, no strategies are, in general, available to restore p53 when it has been genetically deleted or mutated [37]. However, targeting the mechanisms that are responsible for p53 inactivation by changes in compartmentalization or aberrant functions may promote p53 reactivation with dramatic consequences for the cancer itself, such as induction of apoptosis, or they may allow restoration of sensitivity to chemotherapy [38,39,40]. In this review, we will focus on mechanisms that are responsible for p53 functional inactivation by changes in compartmentalization or by protein interactions, and on strategies to reactivate p53. In particular, we will describe the role of IκB-α as a potentially relevant p53 partner.

## 2. p53 Functional Inactivation

### 2.1. Mechanisms of p53 Shuttling

Aberrant tumor suppressor compartmentalization is a known mechanism of functional inactivation for various tumor suppressors [41,42]. Tumor suppressors may shuttle, as a consequence of point mutations (such as NPM1 in acute myeloid leukemia [43]) or aberrant regulations [42]. Notably, the mechanisms of nucleo-cytoplasmic transport offer challenging opportunities as a therapeutic target in various cancers [44]. This last mechanisms is, indeed, the most frequent cause of protein shuttling, as described for FoxO [45,46], PTEN [47], p27 [48], and many others [41]. The p53 protein is also a nuclear-cytoplam shuttling protein, due to the presence of two nuclear export signals (NES), one located within the C-terminal oligomerization domain and one in the N terminus, as well as a nuclear localization signal (NLS) at its C-terminal domain [49,50]. p53 shuttling is also modulated by mono-ubiquitination, while poly-ubiquitination is responsible of p53 degradation. Mono-ubiquitination of p53 promotes its translocation from the nucleus to the cytoplasm [51,52,53,54]. Notably, while Mdm2 is the E3 ligase promoting p53 poly-ubiquitination, the contribution of Mdm-2 in mediating p53 mono-ubiquitination is much more complex [55]. In particular, it was demonstrated that low levels of Mdm2 activity dictate p53 mono-ubiquitination, while high levels promote polyubiquitination [53]. To increase the complexity of the mechanisms of regulation of p53 delocalization by ubiquitination, it should be noted that other enzymes are involved in p53 monoubiquitination. In particular, the E3 ligase, Msl2, was shown to mediate p53 monoubiquitination, and subsequent shuttling into the cytoplasms, in a Mdm2-independent manner [56]. Notably, Msl2 is not involved in the degradation of p53. Similarly, the NEDD4-like WWP1 E3 ligase was shown to promote p53 nuclear export, in a Mdm2 dispensable manner [57]. p53 ubiquitination is counteracted by the de-ubiquitinase HAUSP, rendering the mechanisms of p53 mono/poly-ubiquitination highly complex [58]. Furthermore, the result of shuttling versus degradation of p53 is a consequence of a dynamic process encompassing many cellular components and, at least at the moment, the development of strategies to modulate p53 localization with specific inhibitors is debatable, but attractive. However, it is clear that the mechanisms that promote p53 nuclear exclusion may also be able to promote the loss of the nuclear tumor suppressive function of p53, with clear implication in tumorigenesis.

### 2.2. p53 Regulatory Proteins

An additional mechanism for p53 functional inactivation is exerted by the activity of various p53-interacting proteins that are able to (i) prevent p53 to function as a transcriptional factor; and (ii) favor aberrant p53 cellular compartmentalization and activity [59]. Among the several p53-interacting proteins, it is worth mentioning the functions of few of them. The Mdmx protein is an essential component of the p53 regulatory machinery, which includes Mdm2 [60]. While Mdm2 acts as an E3 ligase, which promotes p53 degradation, the Mdmx protein physically interacts with the transcriptional domain of p53, preventing it from mediating gene expression. Mdmx/p53 is an example of how genetically wild-type p53 can be functionally inactivated by interaction with a partner. Similarly, the Tax protein was shown to interfere with the transactivation function of p53 in adult T cell leukemia, and, in particular, in HTLV-I transformed cells [61,62]. Other partners, such as E2F [63], NPM [64], and many others, reviewed elsewhere [59], play an essential role in regulating p53 functionality. However, due to the complexity of such regulations, it is difficult to precisely determine the role of these interactions in cancer pathogenesis.

It should also be noted that *TP53* mutations may affect the landscape of p53-interacting proteins, with consequent aberrant and novel gains or losses in functions [1,18,65,66,67]. In particular, p53 mutants can interact with a different set of proteins, such as Tap63, compared to wild-type p53 [68,69], or may also affect downstream targets in a complete different manner when compared to normal p53, as was recently observed with p21WAF1 [70]. The landscape of wild-type and mutant p53 partners is indeed expanding dramatically [25], rendering the study of the biological relevance of all these new networks highly complex, but also offering new opportunities to target cancers with mutant p53.

## 3. The IκB-α/p53 Connection

p53 activity is known to be intimately connected with NF-κB signaling [71,72]. In particular, NF-κB/p53 crosstalk has been associated with various phases of tumorigenesis, including transformation, metastatization, and immunological surveillance. It was demonstrated that NF-κB may either antagonize or cooperate with p53 [73]. Among the different mechanisms, the NF-κB/p53 connection appeared to be dependent on IKK kinase [73,74], which is also involved in the phosphorylation and subsequent degradation of IκB-α.

**Figure 2:**
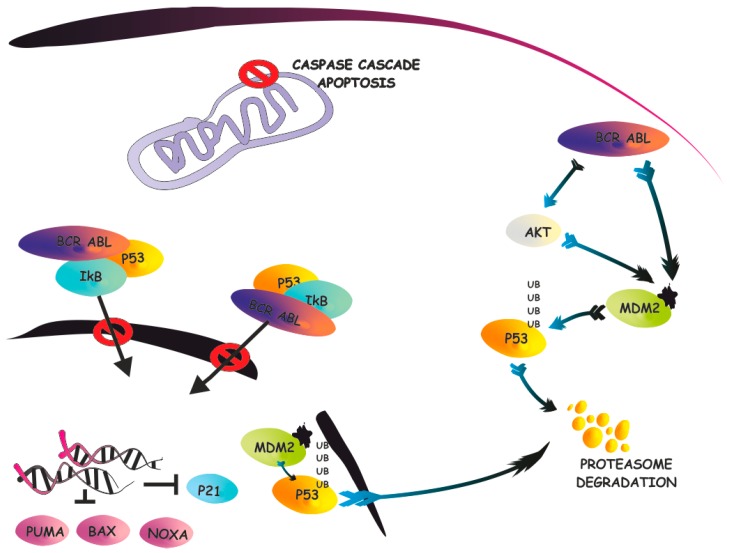
IκB-α/p53 network in chronic myeloid leukemia (CML). IκB-α/p53 network is summarized in the context of CML. In BCR-ABL leukemia cells, IκB-α interacts with BCR-ABL and with p53. The BCR-ABL/IκB-α complex prevents p53 translocation into the nucleus and inhibits its tumor suppressive functions. Activation or “transition-states” such as phosphorilation or ubiquitination, are shown in arrowhead; Inhibition is represented as an arrow with transversal bar.

Notably, IκB-α, the product of the *NFKBIA* gene, was also described as a p53-interacting protein able to modulate p53 functions. IκB-α is mostly renowned as the inhibitor of NF-κB, due to its ability to bind to the p65/p50 dimers, preventing them from translocating into the nucleus, therefore, counteracting NF-κB signaling [75,76]. Upon stimulation, IκB-α is degraded by the proteasome, enabling NF-κB to shuttle into the nucleus where it acts as a transcriptional factor. IκB-α is also known in the clinical environment because the proteasome inhibitor bortezomib, routinely used to treat multiple myeloma, is able to prevent IκB-α degradation, therefore blocking NF-κB signaling [77]. A few manuscripts have been published that demonstrate that IκB-α is, not only able to modulate NF-κB signaling, but is also able to promote p53 functional inactivation [78,79,80,81]. In an original report, it was shown that IκB-α/p53 is formed, both in the cytoplasm, and in the nucleus under basal conditions, and is dissociated in response to apoptotic stress, DNA damage, hypoxia, and TGF-β stimulation [78]. A yeast two-hybrid system allowed mapping of the interaction sites involved in the binding of p53. In particular, the non-ankyrin C terminus of IκB-α interacts with the proline-rich region of p53, while the phosphorylation of p53 at ser46 is involved in the regulation of the interaction. This interaction was also confirmed by another group that demonstrated that the protein structure of p53 is similar to that of the p65 subunit of NF-κB [80]. Following this evidence, the authors claimed, and demonstrated, that IκB-α is able to bind to both p65 and p53, and, in both cases, this interaction inactivates the partners. These two works, however, lead to two partially different conclusions. One demonstrated that the interaction of IκB/p53 suppresses p53 activity, while, in the other, the exogenous co-expression of both proteins was associated with increased apoptosis induction. This discrepancy may be explained by the experimental approaches, since exogenous expression of proteins might not reproduce physiological processes. A third work was subsequently published, clearly demonstrating that, when IκB-α is mutated on the two serine residues that dictate its degradation, IκB-α is stable in the cytoplasm and is bound to both p53 and p65 [81]. As a consequence, the transcriptional activities of both p65 and p53 are suppressed. This observation was also consistent with the fact that expression of this mutant form of IκB-α was shown to suppress p53-dependent apoptosis in lymphoblastic leukemia, as demonstrated by another group [79]. The removal of the ankyrin repeated domain and the NES domain in IκB-α promotes the location of IκB-α into the nucleus, where it was shown to promote both p65 and p53 transcriptional activities. Again, while the use of mutants may affect the physiological function of the IκB-α/p53 interaction, this work further supports the notion that IκB-α/p53 is a potentially-relevant, novel network that is involved in the functional inactivation of p53. It should also be noted that NF-κB is able to directly regulate p53 and vice versa, and, therefore, the net result of p53 regulation by IκB-α mutants may not necessary recapitulate the direct interaction with p53. In addition to the experimental evidence that supports the IκB-α/p53 complex, it is also needed to investigate whether this network is important in cancer specimens. Recently, we observed that, in chronic myeloid leukemia (CML), a myeloproliferative disorder sustained by the t(9:22) translocation coding for the chimeric protein BCR-ABL [82,83], the IκB-α protein is highly expressed in the cytoplasm [84]. Notably, we also documented that cytoplasmic IκB-α is bound to p53 in primary CML cells (Figure 2). Interestingly, this interaction does not appear to be mediated by tyrosine phosphorylation of IκB-α by BCR-ABL, as IκB-α was not found to be tyrosine-phosphorylated by BCR-ABL in primary cells and in in vitro kinase assays. Therefore, we speculated that BCR-ABL simply physically interacts with IκB-α, and that this event may favor some conformation changes or other modifications in IκB-α, which, in turn, promotes interaction with p53. As a consequence, p53 is excluded from the nucleus and loses its nuclear tumor suppressive role. We have observed that p21, a p53 target, is significantly down-modulated in CML and is restored to normal levels upon treatment with imatinib, the BCR-ABL inhibitor [84]. Interestingly, very recent work has demonstrated that p53 targets are downregulated in CML. While this observation may be coherent with our data [85], in this manuscript, p53 did not appear to be excluded from the nucleus. It could be possible that p53 nuclear exclusion, as observed for PTEN compartmentalization in CML [47], is dependent on the differentiation status of the cells. In the case of PTEN, for instance, only progenitor cells are characterized by PTEN nuclear exclusion, while CML stem cells retain physiologically diffuse localization [47]. It is possible, and should be experimentally investigated, that p53 cellular compartmentalization is different in CML progenitors versus primitive CML CD34^+^ CD38^−^ stem cells. Notably, this process appears to be therapeutically relevant because treatment with imatinib promoted p53 such that it shuttled back into the nucleus [84]. Further analyses should be performed to assess whether p53 is trapped in the cytoplasm by IκB-α in challenging Philadelphia positive scenarios, such Ph^+^-ALL or Tyrosine Kinase Inhibitor (TKI)-resistant CML. In these contexts, restoring p53 into the nucleus may have profound consequences and may favor leukemia eradication.

## 4. Targeting the IκB-α/p53 Network

Various works have clearly demonstrated that IκB-α and p53 are part of a novel network [78,79,80,81,84]. Targeting upstream proteins, such as BCR-ABL, favors the restoration of p53 into the nucleus, as we noted. Thus, strategies that aim at disrupting the IκB-α/p53 network would favor p53 nuclear pool restoration, specifically in contexts where BCR-ABL inhibition is no longer sufficient to promote cancer apoptosis induction. Currently, no strategies to disrupt IκB-α/p53 interaction have been described and, therefore, here, we simply speculate on the effects of the clinically-available IKK inhibitors and proteasome inhibitors on this complex. IKK inhibitors block the phosphorylation of IκB-α, which is indeed unable to be degraded, since phosphorylation primes IκB-α to ubiquitination, while proteasome inhibitors indirectly stabilize IκB-α, blocking proteasome activity. In the absence of data, it is debatable to speculate on the consequences of these drugs on the IκB-α/p53 complex because, as is well documented, these drugs significantly impact NF-κB function, and the final biological consequences may be a balancing of effects toward p53 and NF-κB. However, IKK inhibitors and IκB-α inhibitors may substantially increase the levels of IκB-α protein, and may favor the generation of the IκB-α/p53 complex. It is possible that acquired resistance to these drugs, or the refractoriness observed in some patients, is the consequence of the trapping of p53 in the cytoplasm, which thwarts NF-κB inhibition. Consequently, a potential therapeutic strategy should be a drug that stabilizes IκB-α, with consequent NF-κB inhibition, and which prevents binding with p53, therefore inducing apoptosis.

## 5. Discussion

The functional inactivation of tumor suppressors, and in particular p53, is an essential novel concept that may offer promising therapeutic opportunities [34,44]. On the one hand, p53 inactivation either through mutations or aberrant regulations may bring out novel targets, as was recently pointed out with the DNA damage pathway and Arf regulation [86]. On the other hand, p53 inactivation allows for the design of novel therapies that are able to promote its re-activation, with the important induction of cancer selective apoptosis. Various tumor suppressors have been shown to be functionally inactive in cancer, such as p27, FoxO, PTEN, and p53. In this work, we have described a few mechanisms that are able to inactivate p53, which include protein shuttling and protein inactivation by p53-interacting partners. Among these partners, we have discussed, in detail, the role of IκB-α, as an essential mediator of either the p53 or p65 subunit of NF-κB. While the interaction has already been demonstrated in various models and contexts, the potential therapeutic relevance is still waiting for a deeper investigation, but may represent a new opportunity to restore p53 functions.

## References

[1] Muller P.A.J., Vousden K.H. (2014). Mutant p53 in cancer: New functions and therapeutic opportunities. Cancer Cell.

[2] Donehower L.A., Lozano G. (2009). 20 years studying p53 functions in genetically engineered mice. Nat. Rev. Cancer.

[3] Levine A.J., Oren M. (2009). The first 30 years of p53: Growing ever more complex. Nat. Rev. Cancer.

[4] Zilfou J.T., Lowe S.W. (2009). Tumor suppressive functions of p53. Cold Spring Harb. Perspect. Biol..

[5] Olivier M., Hollstein M., Hainaut P. (2010). *TP53* mutations in human cancers: Origins, consequences, and clinical use. Cold Spring Harb. Perspect. Biol..

[6] Petitjean A., Achatz M.I.W., Borresen-Dale A.L., Hainaut P., Olivier M. (2007). *TP53* mutations in human cancers: Functional selection and impact on cancer prognosis and outcomes. Oncogene.

[7] Joerger A.C., Fersht A.R. (2008). Structural biology of the tumor suppressor p53. Annu. Rev. Biochem..

[8] Soussi T., Wiman K.G. (2007). Shaping genetic alterations in human cancer: The p53 mutation paradigm. Cancer Cell.

[9] Purvis J.E., Karhohs K.W., Mock C., Batchelor E., Loewer A., Lahav G. (2012). p53 dynamics control cell fate. Science.

[10] Stracquadanio G., Wang X., Wallace M.D., Grawenda A.M., Zhang P., Hewitt J., Zeron-Medina J., Castro-Giner F., Tomlinson I.P., Goding C.R. (2016). The importance of p53 pathway genetics in inherited and somatic cancer genomes. Nat. Rev. Cancer.

[11] Li T., Kon N., Jiang L., Tan M., Ludwig T., Zhao Y., Baer R., Gu W. (2012). Tumor suppression in the absence of p53-mediated cell-cycle arrest, apoptosis, and senescence. Cell.

[12] Prives C., Lowe S.W. (2015). Cancer: Mutant p53 and chromatin regulation. Nature.

[13] Bieging K.T., Mello S.S., Attardi L.D. (2014). Unravelling mechanisms of p53-mediated tumour suppression. Nat. Rev. Cancer.

[14] Brown C.J., Lain S., Verma C.S., Fersht A.R., Lane D.P. (2009). Awakening guardian angels: Drugging the p53 pathway. Nat. Rev. Cancer.

[15] Vassilev L.T., Vu B.T., Graves B., Carvajal D., Podlaski F., Filipovic Z., Kong N., Kammlott U., Lukacs C., Klein C. (2004). In vivo activation of the p53 pathway by small-molecule antagonists of MDM2. Science.

[16] Khoo K.H., Hoe K.K., Verma C.S., Lane D.P. (2014). Drugging the p53 pathway: Understanding the route to clinical efficacy. Nat. Rev. Drug Discov..

[17] Mello S.S., Attardi L.D. (2013). Not all p53 gain-of-function mutants are created equal. Cell Death Differ..

[18] Abraham C.G., Espinosa J.M. (2015). The crusade against mutant p53: Does the COMPASS point to the Holy Grail?. Cancer Cell.

[19] Zhu J., Sammons M.A., Donahue G., Dou Z., Vedadi M., Getlik M., Barsyte-Lovejoy D., Al-awar R., Katona B.W., Shilatifard A. (2015). Gain-of-function p53 mutants co-opt chromatin pathways to drive cancer growth. Nature.

[20] Brosh R., Rotter V. (2009). When mutants gain new powers: News from the mutant p53 field. Nat. Rev. Cancer.

[21] Li M., He Y., Feng X., Huang J. (2012). Genome-wide studies of the transcriptional regulation by p53. Biochim. Biophys. Acta.

[22] Strano S., Dell’Orso S., Di Agostino S., Fontemaggi G., Sacchi A., Blandino G. (2007). Mutant p53: An oncogenic transcription factor. Oncogene.

[23] Riley T., Sontag E., Chen P., Levine A. (2008). Transcriptional control of human p53-regulated genes. Nat. Rev. Mol. Cell Biol..

[24] Bieging K.T., Attardi L.D. (2012). Deconstructing p53 transcriptional networks in tumor suppression. Trends Cell Biol..

[25] Menendez D., Inga A., Resnick M.A. (2009). The expanding universe of p53 targets. Nat. Rev. Cancer.

[26] Green D.R., Kroemer G. (2009). Cytoplasmic functions of the tumour suppressor p53. Nature.

[27] Kamp W.M., Wang P.Y., Hwang P.M. (2016). *TP53* mutation, mitochondria and cancer. Curr. Opin. Genet. Dev..

[28] Comel A., Sorrentino G., Capaci V., del Sal G. (2014). The cytoplasmic side of p53’s oncosuppressive activities. FEBS Lett..

[29] Nguyen T.A., Menendez D., Resnick M.A., Anderson C.W. (2014). Mutant *TP53* posttranslational modifications: Challenges and opportunities. Hum. Mutat..

[30] Bode A.M., Dong Z. (2004). Post-translational modification of p53 in tumorigenesis. Nat. Rev. Cancer.

[31] Dai C., Gu W. (2010). p53 post-translational modification: Deregulated in tumorigenesis. Trends Mol. Med..

[32] Lujambio A., Akkari L., Simon J., Grace D., Tschaharganeh D.F., Bolden J.E., Zhao Z., Thapar V., Joyce J.A., Krizhanovsky V. (2013). Non-cell-autonomous tumor suppression by p53. Cell.

[33] Hock A.K., Vousden K.H. (2012). Tumor suppression by p53: Fall of the triumvirate?. Cell.

[34] Selivanova G. (2014). Wild type p53 reactivation: From lab bench to clinic. FEBS Lett..

[35] Ventura A., Kirsch D.G., McLaughlin M.E., Tuveson D.A., Grimm J., Lintault L., Newman J., Reczek E.E., Weissleder R., Jacks T. (2007). Restoration of p53 function leads to tumour regression in vivo. Nature.

[36] Martins C.P., Brown-Swigart L., Evan G.I. (2006). Modeling the therapeutic efficacy of p53 restoration in tumors. Cell.

[37] Mandinova A., Lee S.W. (2011). The p53 pathway as a target in cancer therapeutics: Obstacles and promise. Sci. Transl. Med..

[38] Blanden A.R., Yu X., Loh S.N., Levine A.J., Carpizo D.R. (2015). Reactivating mutant p53 using small molecules as zinc metallochaperones: Awakening a sleeping giant in cancer. Drug Discov. Today.

[39] Paek A.L., Liu J.C., Loewer A., Forrester W.C., Lahav G. (2016). Cell-to-cell variation in p53 dynamics leads to fractional killing. Cell.

[40] Hupp T.R., Hayward R.L., Vojtesek B. (2012). Strategies for p53 reactivation in human sarcoma. Cancer Cell.

[41] Fabbro M., Henderson B.R. (2003). Regulation of tumor suppressors by nuclear-cytoplasmic shuttling. Exp. Cell Res..

[42] Salmena L., Pandolfi P.P. (2007). Changing venues for tumour suppression: Balancing destruction and localization by monoubiquitylation. Nat. Rev. Cancer.

[43] Falini B., Mecucci C., Tiacci E., Alcalay M., Rosati R., Pasqualucci L., La Starza R., Diverio D., Colombo E., Santucci A. (2005). Cytoplasmic nucleophosmin in acute myelogenous leukemia with a normal karyotype. N. Engl. J. Med..

[44] Gravina G.L., Senapedis W., McCauley D., Baloglu E., Shacham S., Festuccia C. (2014). Nucleo-cytoplasmic transport as a therapeutic target of cancer. J. Hematol. Oncol..

[45] Naka K., Hoshii T., Muraguchi T., Tadokoro Y., Ooshio T., Kondo Y., Nakao S., Motoyama N., Hirao A. (2010). TGF-β-FOXO signalling maintains leukaemia-initiating cells in chronic myeloid leukaemia. Nature.

[46] Pellicano F., Scott M.T., Helgason G.V., Hopcroft L.E.M., Allan E.K., Aspinall-O’Dea M., Copland M., Pierce A., Huntly B.J.P. (2014). The antiproliferative activity of kinase inhibitors in chronic myeloid leukemia cells is mediated by FOXO transcription factors. Stem Cells.

[47] Morotti A., Panuzzo C., Crivellaro S., Pergolizzi B., Familiari U., Berger A.H., Saglio G., Pandolfi P.P. (2014). BCR-ABL disrupts PTEN nuclear-cytoplasmic shuttling through phosphorylation-dependent activation of HAUSP. Leukemia.

[48] Agarwal A., Mackenzie R.J., Besson A., Jeng S., Carey A., LaTocha D.H., Fleischman A.G., Duquesnes N., Eide C.A., Vasudevan K.B. (2014). BCR-ABL1 promotes leukemia by converting p27 into a cytoplasmic oncoprotein. Blood.

[49] Nie L., Sasaki M., Maki C.G. (2007). Regulation of p53 nuclear export through sequential changes in conformation and ubiquitination. J. Biol. Chem..

[50] O’Keefe K., Li H., Zhang Y. (2003). Nucleocytoplasmic shuttling of p53 is essential for MDM2-mediated cytoplasmic degradation but not ubiquitination. Mol. Cell. Biol..

[51] Brooks C.L., Gu W. (2004). Dynamics in the p53-Mdm2 ubiquitination pathway. Cell Cycle.

[52] Brooks C.L., Li M., Gu W. (2004). Monoubiquitination: The signal for p53 nuclear export?. Cell Cycle.

[53] Li M., Brooks C.L., Wu-Baer F., Chen D., Baer R., Gu W. (2003). Mono-versus polyubiquitination: Differential control of p53 fate by Mdm2. Science.

[54] Brooks C.L., Gu W. (2011). p53 regulation by ubiquitin. FEBS Lett..

[55] Hock A.K., Vousden K.H. (2014). The role of ubiquitin modification in the regulation of p53. Biochim. Biophys. Acta.

[56] Kruse J.P., Gu W. (2009). MSL2 promotes Mdm2-independent cytoplasmic localization of p53. J. Biol. Chem..

[57] Laine A., Ronai Z. (2007). Regulation of p53 localization and transcription by the HECT domain E3 ligase WWP1. Oncogene.

[58] Brooks C.L., Li M., Hu M., Shi Y., Gu W. (2007). The p53-Mdm2-HAUSP complex is involved in p53 stabilization by HAUSP. Oncogene.

[59] Braithwaite A.W., del Sal G., Lu X. (2006). Some p53-binding proteins that can function as arbiters of life and death. Cell Death Differ..

[60] Wade M., Li Y.C., Wahl G.M. (2013). MDM2, MDMX and p53 in oncogenesis and cancer therapy. Nat. Rev. Cancer.

[61] Tabakin-Fix Y., Azran I., Schavinky-Khrapunsky Y., Levy O., Aboud M. (2006). Functional inactivation of p53 by human T-cell leukemia virus type 1 Tax protein: Mechanisms and clinical implications. Carcinogenesis.

[62] Pise-Masison C.A., Mahieux R., Radonovich M., Jiang H., Duvall J., Guillerm C., Brady J.N. (2000). Insights into the molecular mechanism of p53 inhibition by HTLV type 1 Tax. AIDS Res. Hum. Retrovir..

[63] Polager S., Ginsberg D. (2009). p53 and E2f: Partners in life and death. Nat. Rev. Cancer.

[64] Kurki S., Peltonen K., Latonen L., Kiviharju T.M., Ojala P.M., Meek D., Laiho M. (2004). Nucleolar protein NPM interacts with HDM2 and protects tumor suppressor protein p53 from HDM2-mediated degradation. Cancer Cell.

[65] Haupt S., Raghu D., Haupt Y. (2016). Mutant p53 drives cancer by subverting multiple tumor suppression pathways. Front. Oncol..

[66] Kim M.P., Zhang Y., Lozano G. (2015). Mutant p53: Multiple mechanisms define biologic activity in cancer. Front. Oncol..

[67] Liu J., Zhang C., Feng Z. (2014). Tumor suppressor p53 and its gain-of-function mutants in cancer. Acta Biochim. Biophys. Sin..

[68] Gaiddon C., Lokshin M., Ahn J., Zhang T., Prives C. (2001). A subset of tumor-derived mutant forms of p53 down-regulate p63 and p73 through a direct interaction with the p53 core domain. Mol. Cell. Biol..

[69] Strano S., Fontemaggi G., Costanzo A., Rizzo M.G., Monti O., Baccarini A., del Sal G., Levrero M., Sacchi A., Oren M. (2002). Physical interaction with human tumor-derived p53 mutants inhibits p63 activities. J. Biol. Chem..

[70] Galanos P., Vougas K., Walter D., Polyzos A., Maya-Mendoza A., Haagensen E.J., Kokkalis A., Roumelioti F.M., Gagos S., Tzetis M. (2016). Chronic p53-independent p21 expression causes genomic instability by deregulating replication licensing. Nat. Cell Biol..

[71] Schneider G., Henrich A., Greiner G., Wolf V., Lovas A., Wieczorek M., Wagner T., Reichardt S., von Werder A., Schmid R.M. (2010). Cross talk between stimulated NF-κB and the tumor suppressor p53. Oncogene.

[72] Schneider G., Krämer O.H. (2011). NFκB/p53 crosstalk—A promising new therapeutic target. Biochim. Biophys. Acta.

[73] Tergaonkar V., Perkins N.D. (2007). p53 and NF-κB crosstalk: IKKα tips the balance. Mol. Cell.

[74] Perkins N.D. (2007). Integrating cell-signalling pathways with NF-κB and IKK function. Nat. Rev. Mol. Cell Biol..

[75] Xia Y., Shen S., Verma I.M. (2014). NF-κB, an active player in human cancers. Cancer Immunol. Res..

[76] Karin M. (2006). Nuclear factor-κB in cancer development and progression. Nature.

[77] Panwalkar A., Verstovsek S., Giles F. (2004). Nuclear factor-κB modulation as a therapeutic approach in hematologic malignancies. Cancer.

[78] Chang N.S. (2002). The non-ankyrin C terminus of IκB-α physically interacts with p53 in vivo and dissociates in response to apoptotic stress, hypoxia, DNA damage, and transforming growth factor-β 1-mediated growth suppression. J. Biol. Chem..

[79] Zhou M., Gu L., Zhu N., Woods W.G., Findley H.W. (2003). Transfection of a dominant-negative mutant NF-κB inhibitor (IκBm) represses p53-dependent apoptosis in acute lymphoblastic leukemia cells: Interaction of IκBm and p53. Oncogene.

[80] Dreyfus D.H., Nagasawa M., Gelfand E.W., Ghoda L.Y. (2005). Modulation of p53 activity by IκB-α: Evidence suggesting a common phylogeny between NF-κB and p53 transcription factors. BMC Immunol..

[81] Li X., Xing D., Wang J., Zhu D.B., Zhang L., Chen X.J., Sun F.Y., Hong A. (2006). Effects of IκB-α and its mutants on NF-κB and p53 signaling pathways. World J. Gastroenterol..

[82] Morotti A., Fava C., Saglio G. (2015). Milestones and monitoring. Curr. Hematol. Malig. Rep..

[83] Saglio G., Morotti A., Mattioli G., Messa E., Giugliano E., Volpe G., Rege-Cambrin G., Cilloni D. (2004). Rational approaches to the design of therapeutics targeting molecular markers: The case of chronic myelogenous leukemia. Ann. N. Y. Acad. Sci..

[84] Crivellaro S., Panuzzo C., Carrà G., Volpengo A., Crasto F., Gottardi E., Familiari U., Papotti M., Torti D., Piazza R. (2015). Non genomic loss of function of tumor suppressors in CML: BCR-ABL promotes IκB-α mediated p53 nuclear exclusion. Oncotarget.

[85] Abraham S.A., Hopcroft L.E.M., Carrick E., Drotar M.E., Dunn K., Williamson A.J.K., Korfi K., Baquero P., Park L.E., Scott M.T. (2016). Dual targeting of p53 and c-MYC selectively eliminates leukaemic stem cells. Nature.

[86] Velimezi G., Liontos M., Vougas K., Roumeliotis T., Bartkova J., Sideridou M., Dereli-Oz A., Kocylowski M., Pateras I.S., Evangelou K. (2013). Functional interplay between the DNA-damage-response kinase ATM and ARF tumour suppressor protein in human cancer. Nat. Cell Biol..

